# 3′,4′-dihydroxyflavonol ameliorates endoplasmic reticulum stress-induced apoptosis and endothelial dysfunction in mice

**DOI:** 10.1038/s41598-018-19584-8

**Published:** 2018-01-29

**Authors:** Yeh Siang Lau, Mohd Rais Mustafa, Ker Woon Choy, Stanley M. H. Chan, Simon Potocnik, Terence P. Herbert, Owen L. Woodman

**Affiliations:** 1 Department of Pharmacology, Faculty of Medicine, University of Malaya, Kuala Lumpur, 50603 Malaysia; 2 School of Health and Biomedical Sciences, RMIT University, Bundoora, VIC 3083 Australia; 3 The College of Science, Joseph Banks Laboratories, University of Lincoln, Green Lane, Lincoln, Lincolnshire LN6 7DL United Kingdom

## Abstract

Endoplasmic reticulum (ER) stress has been implicated in the development of hypertension 3 through the induction of endothelial impairment. As 3′,4′-dihydroxyflavonol (DiOHF) 4 reduces vascular injury caused by ischaemia/reperfusion or diabetes, and flavonols have been demonstrated to attenuate ER stress, we investigated whether DiOHF can protect mice from ER stress-induced endothelial dysfunction. Male C57BLK/6 J mice were injected with tunicamycin to induce ER stress in the presence or absence of either DiOHF or tauroursodeoxycholic acid (TUDCA), an inhibitor of ER stress. Tunicamycin elevated blood pressure and impaired endothelium-dependent relaxation. Moreover, in aortae there was evidence of ER stress, oxidative stress and reduced NO production. This was coincident with increased NOX2 expression and reduced phosphorylation of endothelial nitric oxide synthase (eNOS) on Ser1176. Importantly, the effects of tunicamycin were significantly ameliorated by DiOHF or TUDCA. DiOHF also inhibited tunicamycin-induced ER stress and apoptosis in cultured human endothelial cells (HUVEC). These results provide evidence that ER stress is likely an important initiator of endothelial dysfunction through the induction of oxidative stress and a reduction in NO synthesis and that DiOHF directly protects against ER stress- induced injury. DiOHF may be useful to prevent ER and oxidative stress to preserve endothelial function, for example in hypertension.

## Introduction

The endoplasmic reticulum (ER) is required for the synthesis of all membrane and secretory proteins. Disturbances in ER homeostasis that perturb protein folding, such as ER calcium depletion, changes in the ER redox state and/or the rate of protein synthesis exceeding the rate of folding, can lead to the accumulation of unfolded proteins within the ER resulting in ER stress^1,2^. This in turn triggers the unfolded protein response (UPR), an adaptive response initiated by the activation of three ER transmembrane proteins i.e. 1) inositol-requiring enzyme 1 (IRE1), 2) protein kinase RNA–like endoplasmic reticulum kinase (PERK), and 3) activating transcription factor 6 (ATF6)^3^. Chronic activation of the UPR has been implicated in a number of human pathologies, including hypertension, through the induction of endothelial dysfunction^4–6^.

The hallmark of endothelial dysfunction is impaired endothelium-dependent vasodilatation. This is due to the attenuated production and release of nitric oxide (NO) and other endothelium-derived relaxing factors (EDRFs), increased production of endothelium-derived contracting factors (EDCFs), and/or reduced bioavailabilty of NO due to increased interaction with superoxide anion O_2_^−^^7,8^. ER stress can cause endothelial dysfunction by downregulating endothelial nitric oxide synthase (eNOS) expression in cells and enhancing oxidative stress^9–11^. Moreover, the ER stress inhibitors, 4-phenyl butyrate (PBA) or tauroursodeoxycholic acid (TUDCA) have proved effective in reducing blood pressure and improving vascular reactivity in angiotensin II (Ang II)- or tunicamycin-infused mice and spontaneously hypertensive rats (SHRs), indicating that ER stress may have been a cause of endothelial dysfunction and thus a key factor for these cardiovascular disorders^5,12^.

The flavonols are a subclass of the flavonoids, naturally occurring polyphenolic compounds that are found in fruits and vegetables, and which can effectively ameliorate endothelial dysfunction in peripheral large arteries^13,14^. 3′,4′-Dihydroxyflavonol (DiOHF) is a synthetic flavonol that not only exhibits antioxidant activity but also has been shown to improve endothelial function in aortae in the presence of oxidative stress^15,16^. DiOHF is a more effective antioxidant and vasorelaxant than a wide range of natural flavones and flavonols including quercetin^17^. Interestingly, the antioxidant property of quercetin had been reported to exert anti-apoptotic activity in endothelial cell culture *in vitro*^18,19^. Furthermore, DiOHF has recently been shown to improve NO-mediated endothelium-dependent relaxations in diabetes by reducing NOX2-dependent superoxide production and attenuating eNOS uncoupling in diabetic rat mesenteric arteries^20^. In addition, previous studies reported that DiOHF effectively reduced oxidative stress-related impairment of cardiovascular function after ischaemia/reperfusion in rats and sheep and in arteries from diabetic rats^15,21,22^. Despite the promising characteristic of DiOHF, no studies have explored the potential vascular protective activity of DiOHF in ER stress-mediated endothelial dysfunction. Therefore, the present research explores, for the first time, the effects of DiOHF in ER stress-induced apoptosis and endothelial dysfunction in mice.

## Results

### DiOHF normalizes blood pressure and endothelial function in tunicamycin treated mice

**Figure 1:**
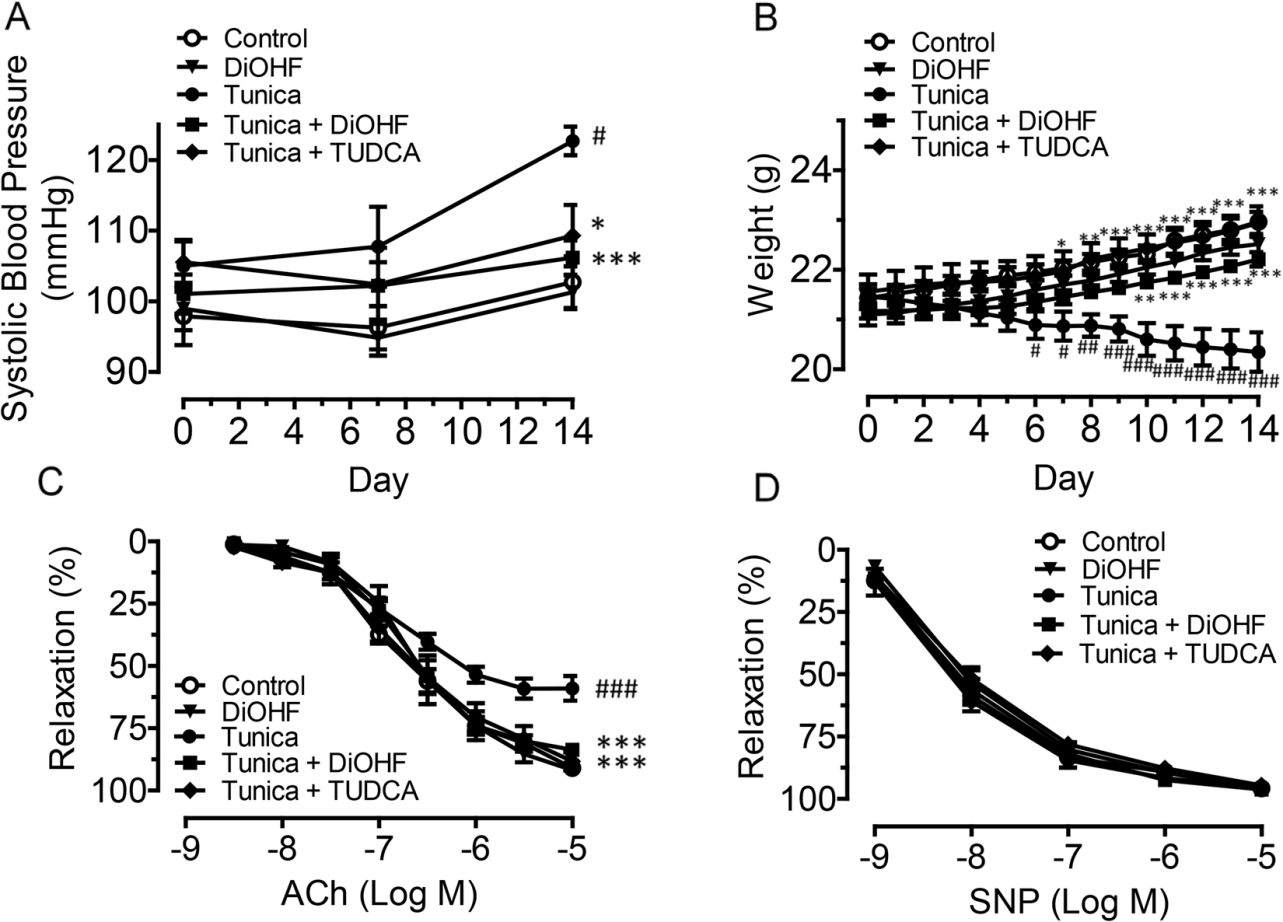
Chronic treatment with DiOHF normalizes systolic blood pressure, body weight and improves endothelial function in mice treated with tunicamycin. (**A**) Systolic blood pressure (SBP) and (**B**) body weight measured in all groups of C57BL/6 J mice that were or were not treated with intra-peritoneal injection of tunicamycin (Tunica, 1 mg/kg, 2 injections/week for two weeks), and the selected group was either treated with DiOHF only (10 mg/kg/day) or treated with tunicamycin plus DiOHF or TUDCA (150 mg/kg/day). (**C**) The impaired ACh-induced endothelium-dependent relaxation was prevented by co-treatment with DiOHF or TUDCA. (**D**) SNP-induced endothelium-independent relaxations in all aortic rings were not affected. Results are mean ± SEM of 6–8 separate experiments. ^#^P < 0.05 vs control, Bonferroni’s Multiple Comparison Test, ^##^P < 0.01 vs control, Bonferroni’s Multiple Comparison Test. ^###^P < 0.001 vs control, Bonferroni’s Multiple Comparison Test. *P < 0.05 vs tunica, Bonferroni’s Multiple Comparison Test. **P < 0.01 vs tunica, Bonferroni’s Multiple Comparison Test. ***P < 0.001 vs tunica, Bonferroni’s Multiple Comparison Test.

To investigate the effect of DiOHF on ER stress-induced endothelial dysfunction, mice were treated for two weeks with tunicamycin, a pharmacological inducer of ER stress, alone or with a co-treatment of DiOHF or TUDCA, a known ER stress inhibitor. A separate group of mice were treated with DiOHF alone to serve as a control group for comparison with the treatment group. Tunicamycin caused a significant increase in systolic blood pressure, 123 ± 2 mmHg compared to untreated controls, 103 ± 4 mmHg. Co-treatment with DiOHF or TUDCA significantly reversed this effect to ~106–109 mmHg for SBP, whereas DiOHF treatment alone had no effect on SBP compared to the untreated control group (Fig. 1A). In addition, the reduced body weight caused by tunicamycin also normalized by co-treatment of DiOHF and TUDCA (Fig. 1B).

Aortae were isolated from these mice and endothelium-dependent and -independent relaxations were assessed using ACh and SNP respectively. Endothelium-dependent relaxation in response to ACh was reduced in isolated aortic rings from tunicamycin-treated mice. This effect was significantly attenuated in mice co-treated with DiOHF or TUDCA (Fig. 1C, Table 1). In contrast, SNP-induced endothelium-independent relaxation was unaffected by tunicamycin (Fig. 1D, Table 1). Thus, tunicamycin caused selective impairment of endothelial function which could be prevented by co-treatment with either DiOHF or the ER stress inhibitor TUDCA.

**Table 1:** Agonist sensitivity (pEC_50_) and % maximum response (R_max_) of endothelium-dependent and -independent relaxations to acetylcholine (ACh) and sodium nitroprusside (SNP) in aortic rings isolated from all treatment groups.

Group	ACh		SNP	
	pEC_50_ (log M)	R_max_ (%)	pEC_50_ (log M)	R_max_ (%)
Control	6.77 ± 0.08	91 ± 1	9.66 ± 1.91	96 ± 1
DiOHF	6.63 ± 0.09	92 ± 2	9.88 ± 3.06	97 ± 1
Tunica	6.83 ± 0.12	59 ± 5^###^	9.55 ± 2.77	96 ± 1
Tunica + DiOHF	6.72 ± 0.05	84 ± 2***	9.04 ± 1.08	96 ± 1
Tunica + TUDCA	6.72 ± 0.05	88 ± 4***	9.23 ± 1.69	95 ± 2

Data are expressed as mean ± SEM (n = 6–8).

^###^P < 0.001 compared vs control.

***P < 0.001 compared vs tunica.

### DiOHF reverses the effects of tunicamycin on ROS and NO bioavailability in *en face* endothelium and aortic rings

**Figure 2:**
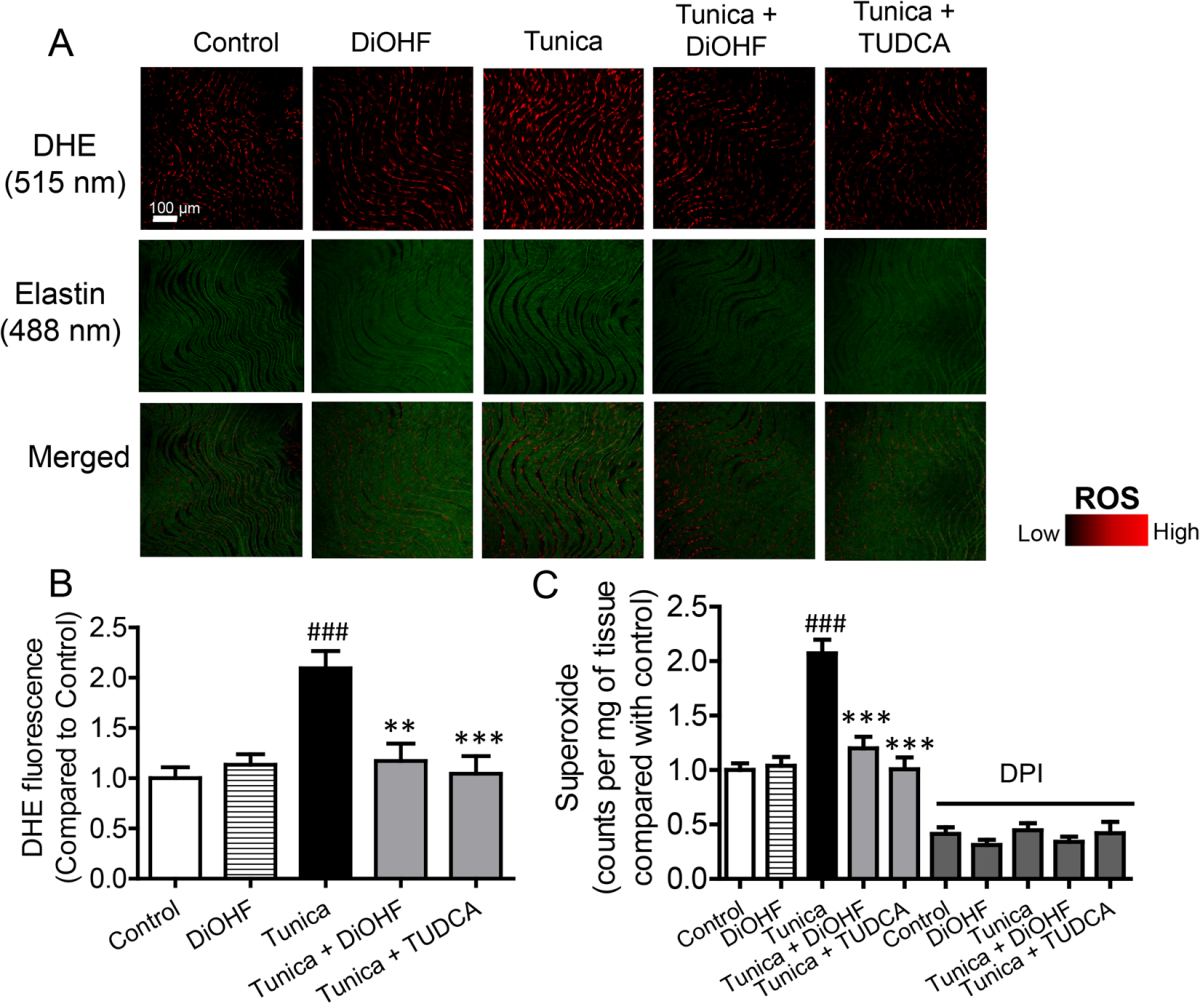
Chronic treatment with DiOHF reduces oxidative stress in mouse aorta. (**A**) Representative images and (**B**) summarized results of ROS measured by DHE fluorescence in the *en face* endothelium of aorta and (**C**) lucigenin-enhanced chemiluminescence assay in the aorta of C57BL/6 J mice. The elevated ROS production in ER stress was reduced by the co-treatment with DiOHF or TUDCA for two weeks. Red: DHE fluorescence (excitation: 515 nm) in the nucleus. Green: autofluorescence of elastin underneath the endothelium (excitation: 488 nm). Lower panel, merged. Bar: 100 μm. Results are mean ± SEM of 6 separate experiments. ^###^P < 0.001 vs control, Bonferroni’s Multiple Comparison Test. **P < 0.01 vs tunica, Bonferroni’s Multiple Comparison Test. ***P < 0.001 vs tunica, Bonferroni’s Multiple Comparison Test.

**Figure 3:**
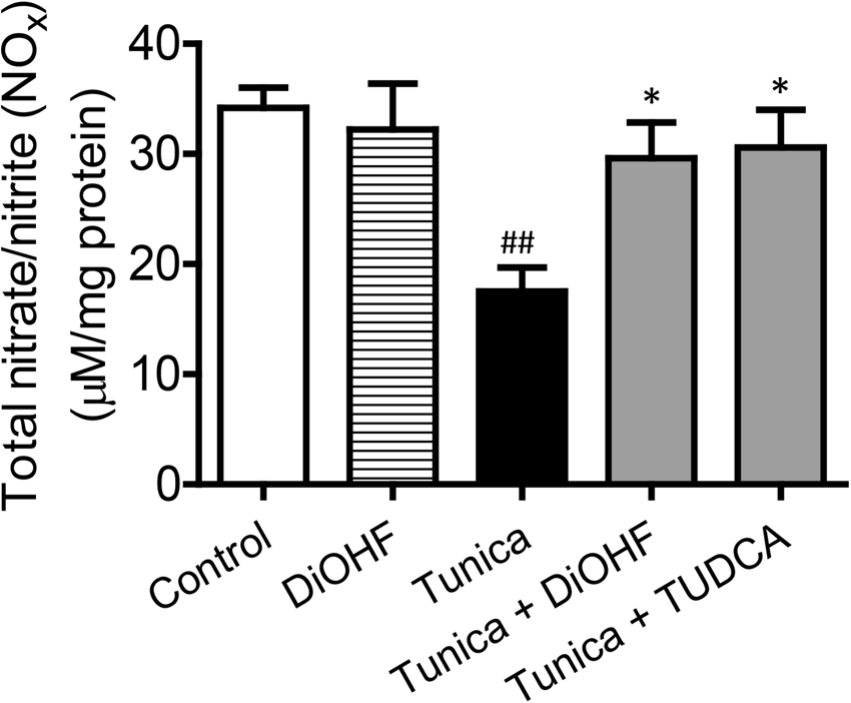
DiOHF treatment for two weeks increased total nitrate/nitrite level. The decreased total nitrite/nitrate levels in ER stress-induced mouse aortae were significantly reversed by the co-treatment of DiOHF and TUDCA. Results are mean ± SEM of 6 separate experiments. ^##^P < 0.01 vs control, Bonferroni’s Multiple Comparison Test. *P < 0.05 vs tunica, Bonferroni’s Multiple Comparison Test.

Increased ROS and decreased NO are characteristics of endothelial dysfunction. Therefore, we investigated changes in vascular oxidative stress and NO in *en face* endothelium and aortic rings isolated from mice treated for 2 weeks with tunicamycin in the presence or absence of DiOHF or TUDCA. Using DHE staining and a lucigenin-enhanced chemiluminescence assay we detected increased ROS levels in tunicamycin-treated mice and this was effectively attenuated by co-treatment with DiOHF or TUDCA (Fig. 2A–C). Nitric oxide (NO) availability was also assessed by measuring its metabolites ie. total nitrate/nitrite levels (NOx). Total NOx levels were reduced in tunicamycin-treated mice but this was prevented by co-treatment with DiOHF or TUDCA (Fig. 3A).

**Figure 4:**
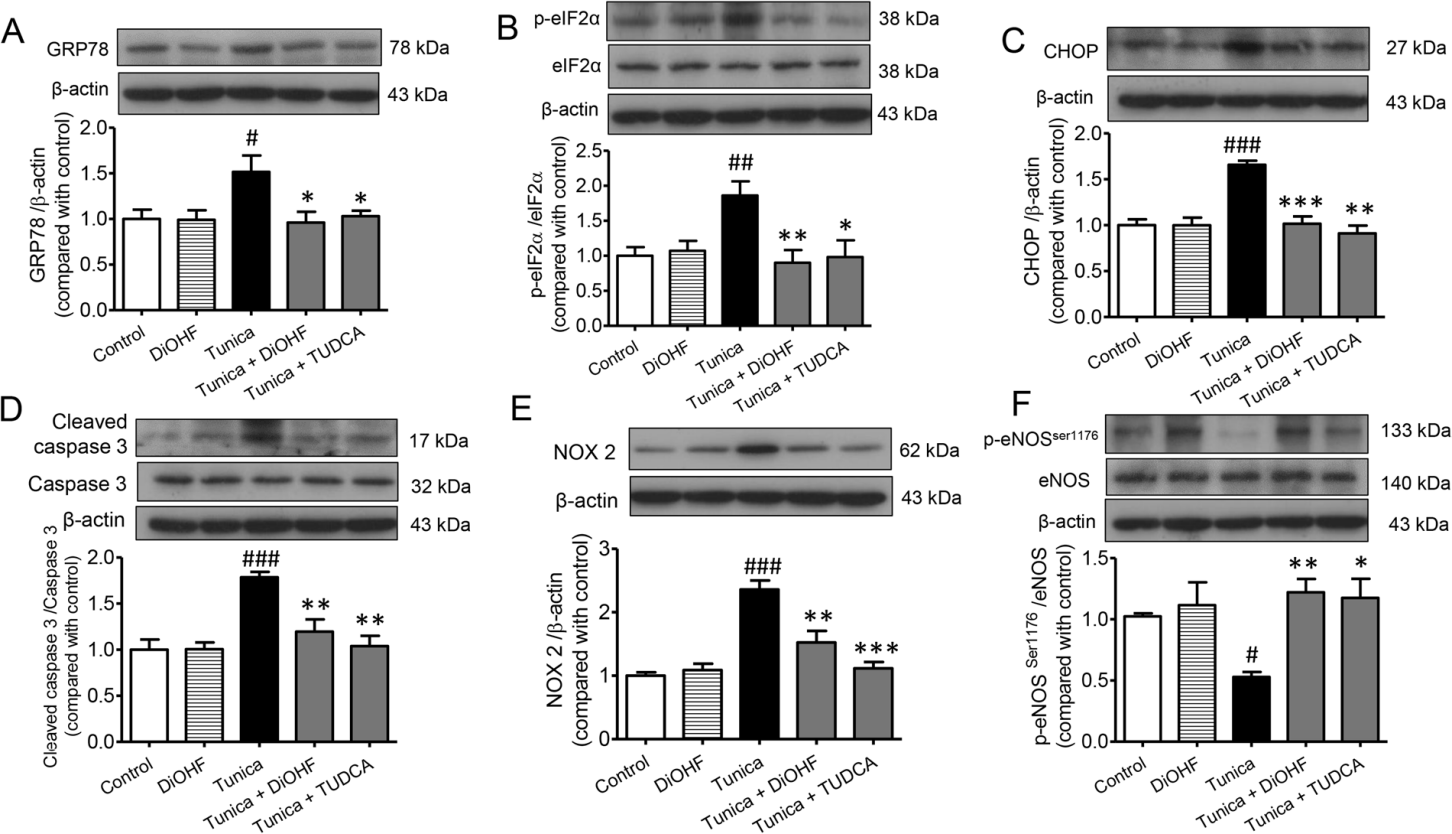
Chronic treatment with DiOHF alleviates ER stress, oxidative stress and apoptosis in mouse aorta. Western blot and quantitative data showing ER stress markers, (**A**) GRP78, (**B**) pelF2α/elF2α; apoptosis markers, (**C**) CHOP, (**D**) cleaved caspase 3/caspase 3; (**E**) oxidative stress marker, NOX2 and (**F**) phosphorylation of eNOS at Ser 1176/eNOS in isolated aortic rings from all treatment groups. Results are mean ± SEM of 6 separate experiments. ^#^P < 0.05 vs control, Bonferroni’s Multiple Comparison Test. ^##^P < 0.01 vs control, Bonferroni’s Multiple Comparison Test. ^###^P < 0.001 vs control, Bonferroni’s Multiple Comparison Test. *P < 0.05 vs tunica, Bonferroni’s Multiple Comparison Test. **P < 0.01 vs tunica, Bonferroni’s Multiple Comparison Test. ***P < 0.001 vs tunica, Bonferroni’s Multiple Comparison Test.

Western blot analysis revealed that NOX2, a subunit of the ROS generator NADPH oxidase, was increased significantly in tunicamycin-treated mice whereas expression of eNOS phosphorylated at Ser1176 (p-eNOS^ser1176^) was found to be decreased, suggesting that the increased oxidative stress reduces eNOS activity. Following DiOHF and TUDCA treatment, protein expression of NOX2 was significantly decreased while p-eNOS^ser1176^ was increased (Fig. 4E and F).

### DiOHF inhibits ER stress and ER stress induced apoptosis in aortic rings isolated from tunicamycin treated mice

To investigate whether DiOHF preserves endothelial function through the alleviation of ER stress in aortic rings isolated from tunicamycin treated mice, we examined the effect of DiOHF on ER stress and apoptosis-associated proteins. As anticipated, the expression of GRP78 and CHOP, as well as the phosphorylation of eIF2α, markers of ER stress, were all increased in aorta isolated from tunicamycin treated mice compared to untreated control mice. Moreover, there was evidence of increased apoptosis in aortae isolated from tunicamycin treated mice compared to untreated control mice as determined by an increase in the abundance of the cleaved form of caspase 3. Importantly, in aortae isolated from mice co-treated with DiOHF or TUDCA, tunicamycin-induced increases in the expression of GRP78 and CHOP, the phosphorylation of eIF2α and the cleaved form of caspase 3, were reduced (Fig. 4A and D). These results provide evidence that DiOHF treatment *in vivo* can inhibit ER stress and ER stress-induced apoptosis in aortic rings.

### DiOHF reduces ER stress and oxidative stress in HUVECs

**Figure 5:**
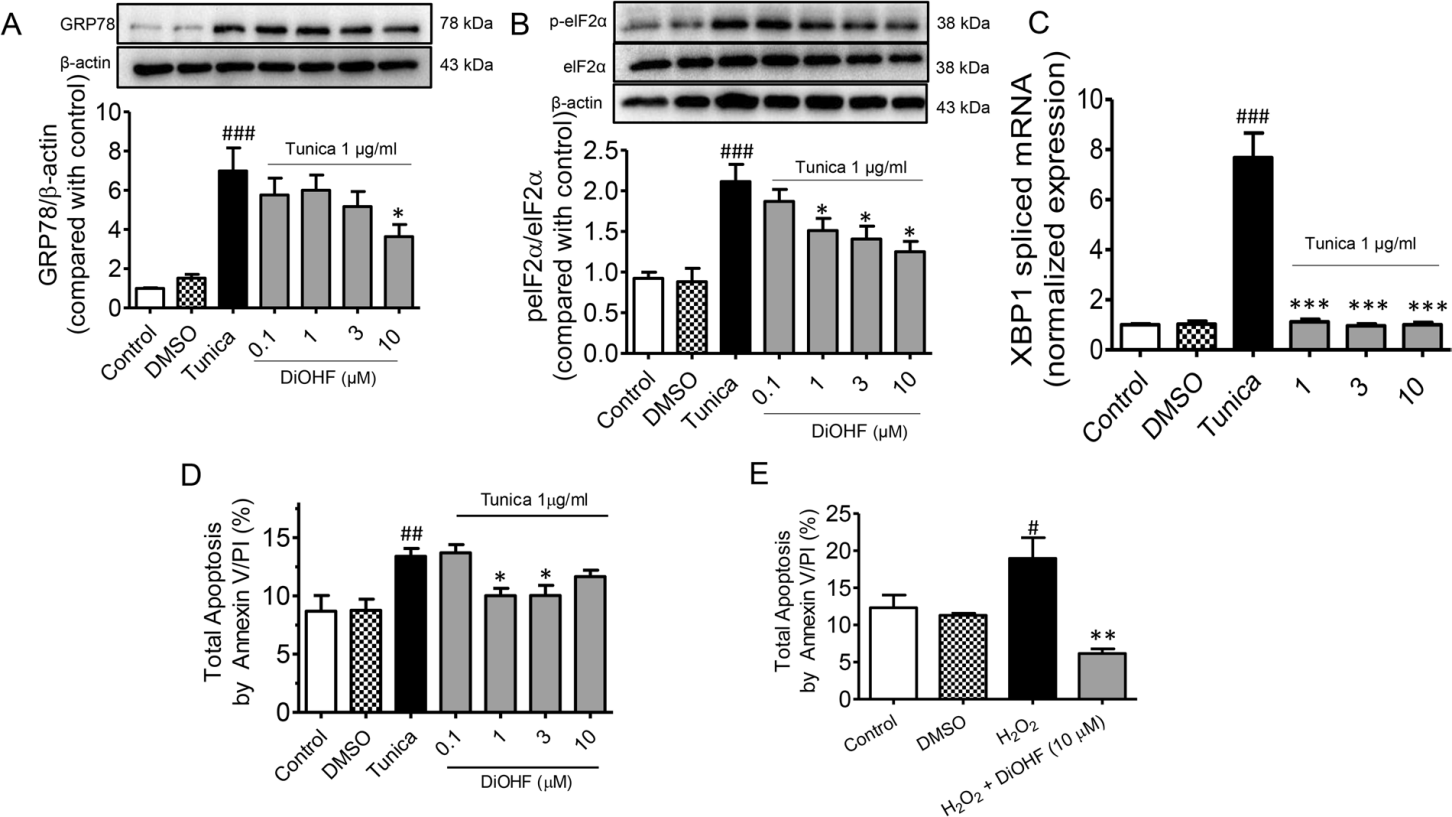
The effect of DiOHF on ER stress and cell apoptosis in HUVECs. The levels of ER stress markers: (**A**) GRP78, (**B**) pelF2α/elF2α, and (**C**) XBP-1 spliced mRNA were measured by western blot and real time-PCR respectively. DiOHF pre-treatment at 10 µM inhibited the upregulation of all 3 ER stress markers while DiOHF (1, 3 and 10 µM) normalized the XBP-1 spliced mRNA similar to DMSO vehicle control. Group data showing DiOHF reduced the number of apoptotic cells induced by 1 µg/ml tunicamycin (**D**) and 200 µM H_2_O_2_ (**E**) in flow cytometry, which was measured by annexin V and propidium iodide (PI) staining. Results are means ± SEM of 3–6 experiments in HUVECs. ^#^P < 0.05 vs control, Bonferroni’s Multiple Comparison Test. ^##^P < 0.01 vs control, Bonferroni’s Multiple Comparison Test. ^###^P < 0.001 vs control, Bonferroni’s Multiple Comparison Test. *P < 0.05 vs tunica, Bonferroni’s Multiple Comparison Test. **P < 0.01 vs tunica, Bonferroni’s Multiple Comparison Test. ***P < 0.001 vs tunica, Bonferroni’s Multiple Comparison Test.

To investigate whether DiOHF could protect against ER stress and ER stress-induced apoptosis *in vitro*, HUVECs were treated with tunicamycin for 16 h in the presence or absence of increasing concentrations of DiOHF and changes in the expression of GRP78, the phosphorylation of eIF2α at Ser52 (p-elF2α), the splicing of XBP-1 mRNA and the extent of apoptosis were determined. Tunicamycin increased GRP78 expression, increased the phosphorylation of eIF2α, caused increased XBP1 splicing and induced apoptosis (Fig. 5A–C). In all cases DiOHF was able to reduce these effects. As DiOHF is a known antioxidant, we examined whether DiOHF could also reduce tunicamycin or H_2_O_2_-induced cell death. Cells were treated with tunicamycin or H_2_O_2_ for 16 h in the presence and absence of DiOHF and the extent of apoptosis determined. Tunicamycin and H_2_O_2_ significantly increased cell death and this effect was significantly reduced by DiOHF (Fig. 5D,E).

## Discussion

In this study, we demonstrated that DiOHF effectively protects the endothelium against ER stress-mediated apoptosis in mice, accompanied by an antioxidant effect. In mice tunicamycin-induced ER stress significantly increased SBP, impaired ACh-induced endothelium-dependent relaxation, caused the up-regulation of ER stress-mediated markers of apoptosis, elevated ROS production and decreased NO bioavailability. Importantly chronic treatment with either DiOHF or the established inhibitor of ER stress, TUDCA, reversed all of the adverse effects indicated above. We show, for the first time, that DiOHF treatment exhibits endothelial protective activity against ER stress-induced injury and dysfunction in mice, and is able to prevent ER stress-induced hypertension. DiOHF has been reported to attenuate cardiovascular dysfunction caused by diabetes and ischaemia^15,20^ and this study indicates for the first time that this flavonol may also protect endothelial function in hypertension.

Compelling evidence has indicated ER stress plays a role in various metabolic and cardiovascular disorders including obesity, hyperglycaemia and hypertension, and that there is associated endothelial dysfunction linked to an increase in ER stress^5,23^. Prolonged ER stress in endothelial cells leads to cell injury, inflammation, apoptosis and ultimately endothelial dysfunction in several chronic diseases^24–26^. The vascular endothelium is important in regulating haemostatic balance and controlling normal vascular tone whereas the impairment of endothelial function may precede and contribute to the development of chronic cardiovascular disorders, particularly in coronary artery disease and other atherosclerotic diseases^27^. Our results are in agreement with previous studies showing that treating mice with tunicamycin leads to an ER stress-induced pre-hypertensive state and endothelial dysfunction in the aorta^5,28^. Recently, ER stress has been implicated in the development of hypertension in several models including Ang II-induced hypertension in mice and in spontaneously hypertensive rats (SHR)^5,11,12^. The studies also showed that treatment with ER stress inhibitors, TUDCA or 4-PBA, effectively ameliorated ER stress, improved endothelial function and lowered blood pressure^5,12^. EDCFs such as cyclooxygenase (COX)-derived prostanoids and increased expression of myosin light chain (MLC_20_) phosphorylated at Ser19 have been implicated as the major vasoconstrictor factors that contributed to ER stress-induced hypertension^12,29^. ER stress inhibitors have been shown to normalize arterial pressure and endothelial function through inhibition of cytosolic phospholipase A2 (cPLA2)/COX pathway and MLC_20_ phosphorylation respectively^6,12^. Similarly, our *in vivo* results demonstrated that co-treatment with DiOHF or TUDCA for two weeks markedly improved ACh-induced endothelium-dependent relaxation and normalized the blood pressure.

Endothelial dysfunction is generally characterized by impaired endothelium-dependent relaxation and decreased NO production often associated with increased oxidative stress^27^. Moreover, prolonged ER stress can lead to the overproduction of ROS from mitochondria and this has been implicated in endothelial dysfunction^30,31^. Our findings indicate that ER stress increased NOX2 protein expression, ROS production and decreased eNOS activity in vascular tissues. Of note, ROS reacts rapidly with NO to promote an oxidative cascade, resulting in reduced NO activity and impaired vascular function in various cardiovascular and metabolic diseases including hypertension and diabetes mellitus^27^. Our study demonstrated that inhibition of ER stress by DiOHF or TUDCA significantly increased NO bioavailability and improved endothelial function in aortae from tunicamycin-treated mice, which may be attributed to the reduction in the level of ROS.

When ER stress occurs, a transient UPR is triggered to relieve ER stress by increasing chaperone proteins, inhibiting protein translation and degrading unfolded proteins^32,33^. A sustained or prolonged exposure to ER stress can lead to programmed cell death, for example, increased CHOP (C/EBP-homologous protein a.k.a growth arrest- and DNA damage inducible gene 153 (GADD153)) expression via activation of PERK and subsequent phosphorylation of eIF2α^32^. A previous study has demonstrated that tunicamycin-induced ER stress led to apoptosis of adipocytes and ultimately reduced body weight which was associated with an increased expression of mRNA for GRP78 and CHOP^34^. We found that tunicamycin injection started to cause a significant weight loss in mice after 6 days of treatment and this effect was reversed by both DiOHF and TUDCA. Furthermore, our results also revealed that tunicamycin treatment in mice leads to an increase in eIF2α phosphorylation, an increase in the expression of CHOP and an increase in caspase 3 activity in isolated mouse aorta, a possible cause of endothelial dysfunction in this model. However, chronic treatment with DiOHF or TUDCA for two weeks significantly reduced ER stress and caspase 3 activity in isolated mouse aorta and restored endothelium-dependent relaxation. Taken together, our results indicate that the protective effect of DiOHF against ER stress-induced endothelial dysfunction is likely through the inhibition of the pelF2α /CHOP/caspase 3 axis. Our findings are consistent with a study by Kassan *et al*. who demonstrated that endothelial dysfunction is associated with the up-regulation of pelF2α/ATF4/CHOP signalling pathway in both aorta and mesenteric resistance arteries (MRA) from hypertensive mice^5^. In addition a report from Kang *et al*. showed that oxidized LDL induced ER stress-mediated endothelial cell apoptosis as a result of an increase in CHOP expression and caspase 3 activity^35^.

**Figure 6:**
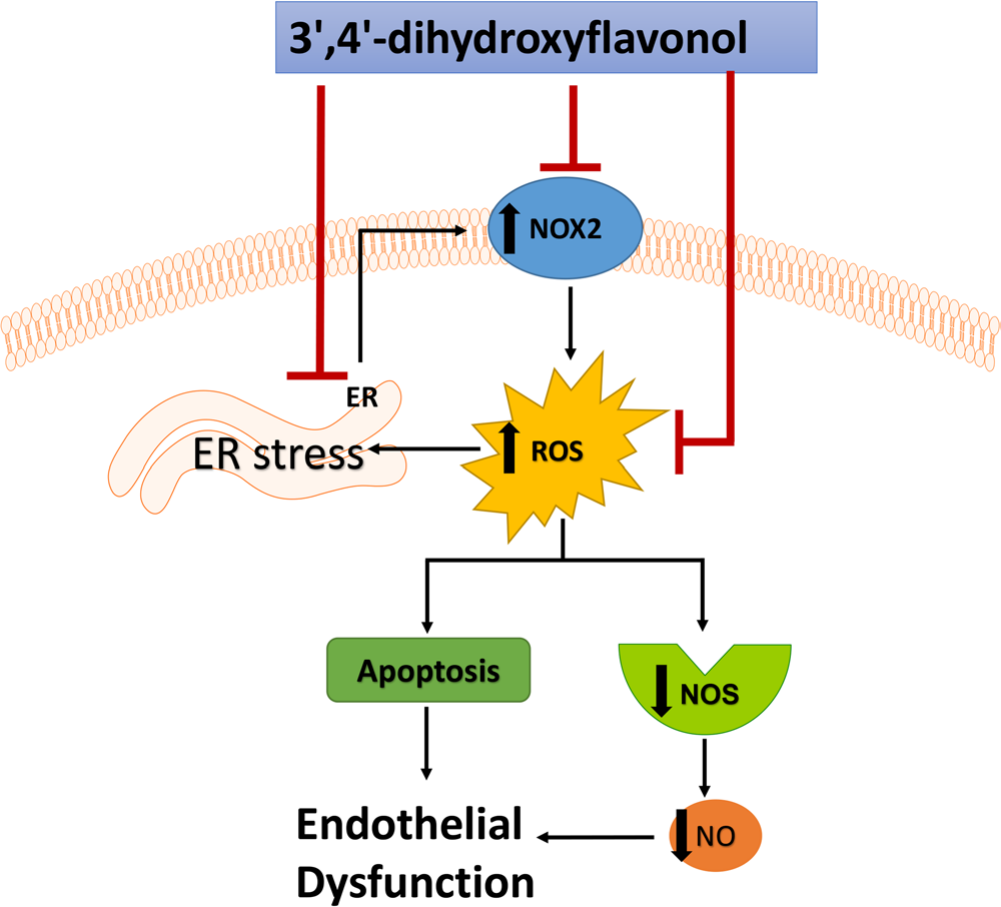
Graphical abstract. DiOHF protects tunicamycin-induced endothelial dysfunction via the inhibition of the ER stress and apoptosis is due at least in part to the alleviation of oxidative stress which, in turn, cause the protein misfolding by interfering with the ER homeostasis.

Several studies have demonstrated that either chemical ER stress inducers, such as tunicamycin and thapsigargin, or physiological inducers of ER stress, such as homocysteine cause ER stress in mammalian cells or animal models^36^. Our data indicates that pre-treatment with DiOHF significantly protected HUVECs from ER stress-induced cell death. Furthermore, DiOHF treatment effectively reduced apoptosis induced by both tunicamycin and H_2_O_2_ demonstrating that DiOHF protects against both ER stress and oxidative stress. Several studies have shown that the activation of ER stress could be reduced by inhibiting the production of intracellular ROS, suggesting an ER stress-dependent oxidative stress signalling pathway^37,38^. Collectively, the protective effect of DiOHF on oxidative stress is exerted, at least in part, via the inhibition of the pelF2α/CHOP/caspase 3 axis, in which oxidative stress promotes the protein misfolding by interfering with the ER homeostasis (Fig. 6).

In conclusion, our study has demonstrated for the first time the therapeutic potential of DiOHF against ER-stress induced apoptosis in both *in vitro* and *in vivo* models. Our findings indicated that DiOHF alleviated ER stress and oxidative stress, which subsequently restored endothelial function in mice by increasing NO bioavailability. Most importantly, our data also showed a lowering of blood pressure in mice after the DiOHF. Hence, our study has provided a new insight into the potential intervention for pre-hypertensive mice by using DiOHF.

## Methods

### Animal model

C57BLK/6 J mice (8 week old males) were purchased from the Monash University Malaysia Campus and all the experimental procedures were approved by the University of Malaya Animal Experimentation Ethics Committee (2016-170531/PHAR/R/MRM). All experiments were performed in accordance with relevant guidelines and regulations and as approved by the ethics committee. Mice were maintained in a well-ventilated room at constant temperature of 24 ± 1 °C and provided with normal rat chow and tap water *ad libitum*. Animals were randomly assigned to 5 groups and received intra-peritoneal injection for two weeks: 1) control group (vehicle, 10% DMSO and 90% peanut oil, 0.2–0.23 ml); 2) DiOHF (10 mg/kg/day); 3) Tunicamycin (Tunica, 1 mg/kg, 2 injections/week); 4) Tunicamycin + DiOHF (Tunica + DiOHF); 5) Tunicamycin + TUDCA (Tunica + TUDCA, 150 mg/kg/day). Body weight was recorded daily and systolic blood pressure (SBP) was recorded weekly using tail-cuff plethysmography (NIBP machine, IITC Inc, Woodland Hills, CA, USA).

### Preparation of vessels and measurement of vascular function

At the end of the treatment period, the mice were sacrificed by CO_2_ inhalation. The aorta was isolated and cleaned of fat and connective tissues. Each aorta was cut into ring segments, 2 mm long and placed in oxygenated Krebs physiological salt solution (KPSS) [composition in mmol/L: NaCl 119, NaHCO_3_ 25, KCl 4.7, KH_2_PO_4_ 1.2, MgSO_4_.7H_2_O 1.2, glucose 11.7, and CaCl_2_.2H_2_O 2.5] and some tissues were snap frozen in liquid nitrogen and stored at −80 °C for later processing. The fresh aortic rings were maintained at 37 °C and stretched to optimal tension of 5 mN in a Multi Wire Myograph System (Danish Myo Technology, Aarhus, Denmark), and continuously oxygenated with 95% O_2_ and 5% CO_2_, and the changes of isometric tension in response to different drugs were recorded using the PowerLab LabChart 6.0 recording system (AD Instruments, Australia).

After 30 min equilibration, the rings were stimulated with 80 mmol/L KCl-containing Krebs solution (KPSS) until a stable contraction was attained and washed with Krebs solution three times before addition of phenylephrine (PE, 3 μmol/L) to obtain a stable contractile response equivalent to 50% of the response to KPSS. Cumulative concentration-response curves to the endothelium-dependent relaxant acetylcholine (ACh, 3 nmol/L to 10 μmol/L) were determined. Endothelium-independent relaxation was tested using sodium nitroprusside, (SNP, 1 nmol/L to 10 μmol/L).

### Measurement of vascular reactive oxygen species (ROS) production in *en face* endothelium and aortic rings

Confocal microscopy was used to examine superoxide generation in the vascular wall of mouse aortae as previous described^39^. The oxidative fluorescent dye dihydroethidium (DHE, 5 μmol/L, Invitrogen) was used to evaluate *in situ* production of superoxide. DHE is freely permeable to the cell membrane and in the presence of superoxide anion is oxidized to ethidium, where it is trapped by intercalating with DNA in the nucleus. The fresh aortic rings were incubated with PBS containing DHE at 37 °C. After 15 min incubation, the excessive DHE was washed away and the aorta placed on a coverslip exposing the luminal surface. The fluorescence intensity at one optical section of the rings was visualized using a confocal microscope Leica TCS SP5 II (Leica Microsystems, Mannheim, Germany) with 515 nm excitation and 585 nm emission and the images were analysed using Leica LAS-AF software version 2.6.0.7266 as represented by the fold change in fluorescence intensity relative to the control mouse aorta. The elastin was captured as autofluorescence at excitation 488 nm and emission 520 nm.

In the second experiment, lucigenin-enhanced chemiluminescence assay was performed based on the work by Chan *et al*.^15^. The aortic rings from all groups were pre-incubated at 37 °C in Krebs-Hepes buffer containing diethylthiocarbamic acid (DETCA, 10 mmol/L) to inactivate superoxide dismutase and β-NADPH (0.3 mmol/L) as a substrate for NADPH oxidase or diphenylene iodonium (DPI) as an inhibitor of NADPH oxidase for 45 minutes. The rings were then transferred to a 96-well plate in a luminometer (Plate CHAMELEONTM, Hidex, Finland). The output of chemiluminescence was measured and the background was subtracted. All of the samples were dried in a 65 °C oven for 48 hours and the result was expressed as counts per milligram dry weight of tissue.

### Total nitrate/nitrite levels

Measurement of total nitrate/nitrite concentration in tissue was measured using a Nitrate/Nitrite Colorimetric Assay Kit (Cayman Chemical Company, USA), according to the manufacturer’s protocol. Absorbance was measured at 540 nm. Nitrite concentrations were calculated by comparison with a standard curve of sodium nitrite.

### Culture of human umbilical vein endothelial cells and treatments

HUVECs were purchased from ScienCell Research Laboratories (Carlsbad, CA, USA). The cells were routinely cultured in endothelial cell medium (ECM) supplemented with 5% FBS, 1% endothelial cell growth supplement, and 1% of penicillin-streptomycin (ScienCell Research Laboratories) at 37 °C in a humidified atmosphere containing 5% CO_2_.

For ER stress induction experiments, the cells were seeded on 6-well plates until they reached 70–80% confluence. The cells were starved in serum-free culture medium for 4 hours prior to treatment. The cells were then pre-treated with different concentrations of DiOHF (0.1–10 µmol/L) for 30 minutes then exposed to the ER stress inducer, tunicamycin (1 µg/ml) for 16 hours. In another set of experiments, H_2_O_2_ (200 µmol/L) was used instead of tunicamycin to investigate the involvement of antioxidant properties of DiOHF in preserving cells from apoptosis.

### Apoptosis assay

The tunicamycin and H_2_O_2_-induced cell death in cells after 16 hours was detected using flow cytometry (BD FACS Canto II, Becton Dickinson). The identification for the early (Annexin V^+^/PI^−^) and late apoptotic (Annexin V^+^/PI^+^) cells were determined by annexin V and propidium iodide (PI) staining from a commercial kit (BD Biosciences, CA, USA) while the necrotic/damaged cells were categorized in Annexin V^−^/PI^+^. Only the cells stained with annexin V^+^ were considered to be apoptotic cells. The assay was performed according to the manufacturer’s instructions and the data analysis was performed with BD FACSDiva™ software, version 6.1.3 (BD Biosciences, Becton Dickinson).

### Real time RT-qPCR assay for XBP1 gene

The assay was performed as described by Schadewijk *et al*.^40^ with the following modification. Briefly, total RNA was extracted using ReliaPrep™ RNA Cell Miniprep Systemp (Promega, USA) then 2 μg RNA was reverse transcribed using High Capacity cDNA Reverse Transcription Kit (Applied Biosystems, UK), according to the manufacturer’s instructions. Quantitative PCR was carried out using the SYBR® Green PCR Master Mix (Applied Biosystems, UK). The XBP1spl forward and reverse primer sequences were 5′TGCTGAGTCCGCAGCAGGTG3′ and 5′GCTGGCAGGCTCTGGGGAAG3′ respectively. Each assay was run on a Rotor-Gene Q (Qiagen, USA) in duplicate and the results are calculated using the relative standard curve method. All gene expressions were normalized against the respective housekeeping gene 18S.

### Western blotting

Cells and mouse aortae harvested from different treatment groups were homogenized in ice-cold 1 X RIPA buffer containing (leupeptin 1 μg/ml, aprotonin 5 μg/ml, PMSF 100 μg/ml, sodium orthovanadate 1 mmol/L, EGTA 1 mmol/L, EDTA 1 mmol/L, NaF 1 mmol/L, and β-glycerolphosphate 2 mg/ml). The lysates were centrifuged at 20,000 g for 10 min and supernatant was collected for Western blotting. The protein concentration of the supernatant was determined using the Lowry assay (Bio-Rad Laboratories, Hercules, CA, USA). Total protein concentration of 10–15 μg for each lane was separated in 10–15% sodium dodecyl sulphate (SDS)-polyacrylamide gel and then transferred to an immobilon-P polyvinylidene difluoride membrane (PVDF, Millipore, Billerica, MA, USA). The blots were blocked for non-specific binding with 3% bovine serum albumin (BSA) in Tris-buffered saline containing 0.1% Tween 20 (TBS) for 1 hour at room temperature with gentle shaking. After rinsing in TBS-T, the blots were incubated at 4 °C overnight with primary antibodies against phosphorylated eIF-2α at Ser^52^ (pelF2α, 1;1000, Cell Signalling Technology, Danvers, MA, USA), total eIF2α (1;1000, Cell Signalling Technology), GRP78 (1:1000, Santa Cruz), CHOP (1;500, Cell Signalling Technology), cleaved caspase 3 (1;500, Cell Signalling Technology), caspase 3 (1;500, Cell Signalling Technology), NOX 2 (1;1000, Abcam, Cambridge, UK), phosphorylated eNOS at Ser 1176 (1;1000, Abcam), and total eNOS (1;1000, BD Transduction laboratory, San Diego, CA, USA). The blots were washed three times for 5 minutes in TBS-T and incubated with respective secondary antibodies conjugated to horseradish peroxidise for 2 hours at room temperature. The blots were developed with a chemiluminescence detection system (ECL reagents, Millipore Corporation, Billerica, MA), and exposed to X-ray films. The images were scanned and densitometric analysis was performed using Quantity One 1D analysis software. The respective protein expression levels were normalized to the housekeeping protein β-actin.

### Chemicals

Tunicamycin, ACh, SNP, phenylephrine, Tween-20, bis-N-methylacridinium nitrate, DETCA, DPI, β-NADPH, Tris-base were purchased from Sigma-Aldrich (St. Louis, MO, USA). DiOHF was purchased from Indofine and dissolved in DMSO. TUDCA was purchased from Calbiochem. Bovine serum albumin (BSA) was purchased from Santa Cruz (Dallas, Texas, USA). Kreb’s salts were purchased from BDH Limited and BDH Laboratory Supplies (Poole, UK).

### Statistical Analysis

Results are presented as mean ± SEM from n mice. Statistical significance were determined using one-way ANOVA or two-way ANOVA as appropriate followed by Bonferroni post hoc-tests whenever appropriate (GraphPad Software, San Diego, USA). A P value of less than 0.05 is regarded as significant.

## Electronic supplementary material

Supplementary Information
