# Supplementary material for: 3′,4′-dihydroxyflavonol ameliorates endoplasmic reticulum stress-induced apoptosis and endothelial dysfunction in mice
Source: Sci Rep. 2018 Jan 29;8:1818. doi: 10.1038/s41598-018-19584-8 (PMC5789000; doi:10.1038/s41598-018-19584-8)
Supplement: Supplementary file 1 — Supplementary Information [file 41598_2018_19584_MOESM1_ESM.pdf]

# 3’,4’-dihydroxyflavonol ameliorates endoplasmic reticulum stress-induced apoptosis and endothelial dysfunction in mice

**Yeh Siang Lau[1], Mohd Rais Mustafa[1], Ker Woon Choy[1], Stanley M.H. Chan[2], Simon Potocnik[2], Terence P. Herbert[2,3], Owen L. Woodman[2]***

[1]Department of Pharmacology, Faculty of Medicine, University of Malaya, Kuala Lumpur 50603, Malaysia;

[2]School of Health and Biomedical Sciences, RMIT University, Bundoora, VIC 3083, Australia

[3]The College of Science, Joseph Banks Laboratories, University of Lincoln. Green Lane, Lincoln, Lincolnshire. LN6 7DL

*Corresponding Author:

Professor Owen L. Woodman

School of Health and Biomedical Sciences, RMIT University, PO Box 71, Bundoora, VIC 3083 Australia

Tel: +61 3 9925 7305

Email: owen.woodman@rmit.edu.au

## Figure 4A

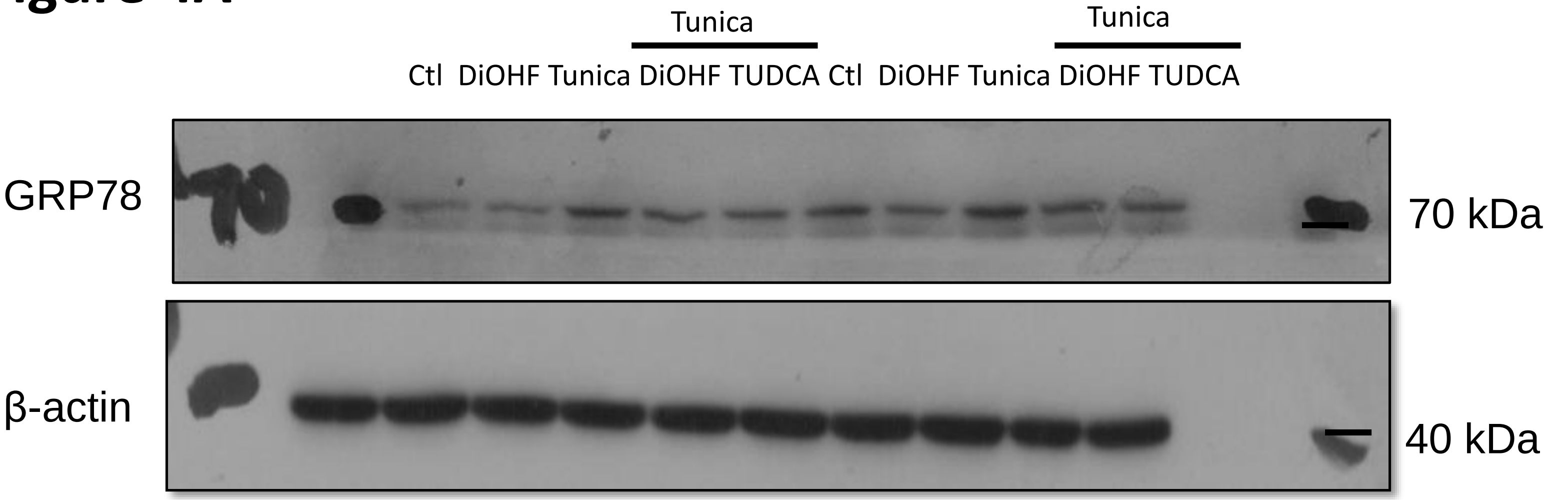


## Figure 4B

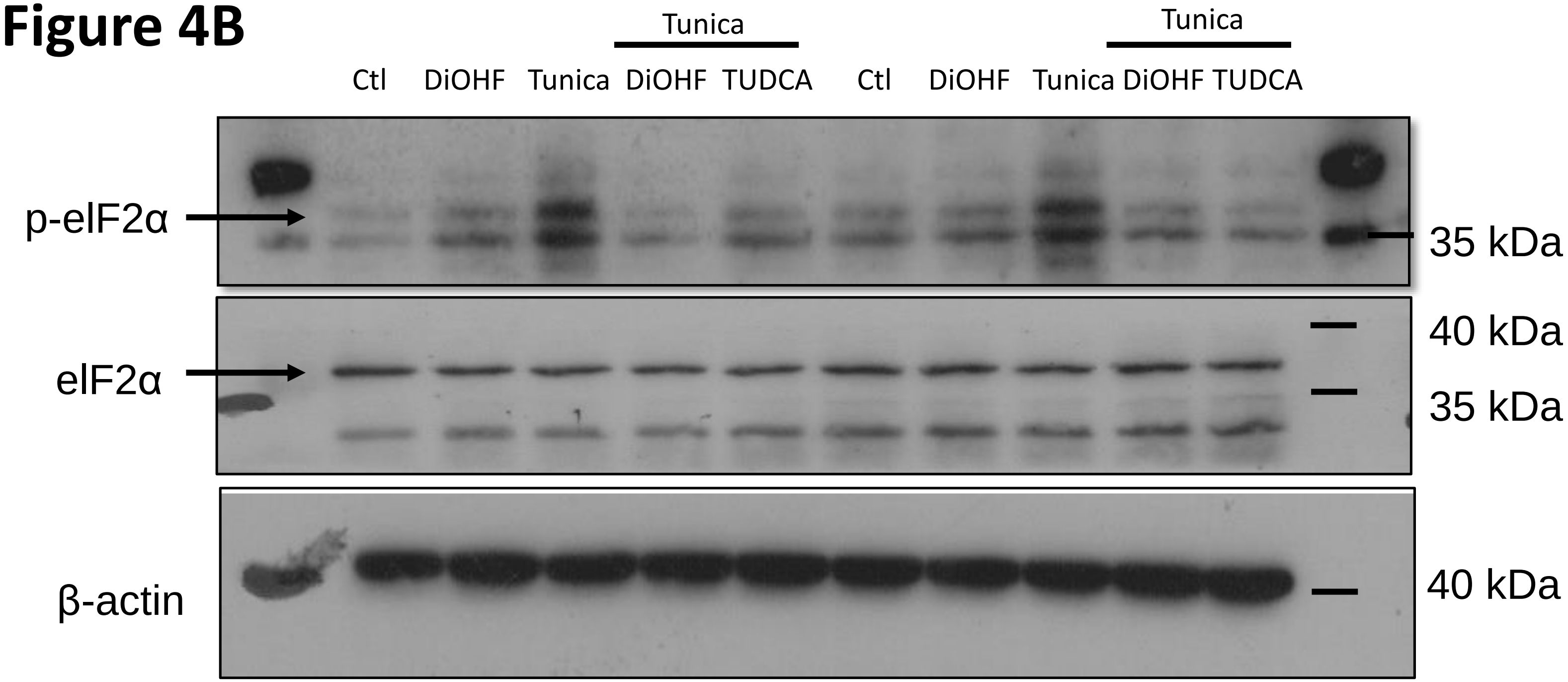

**Figure 4C**

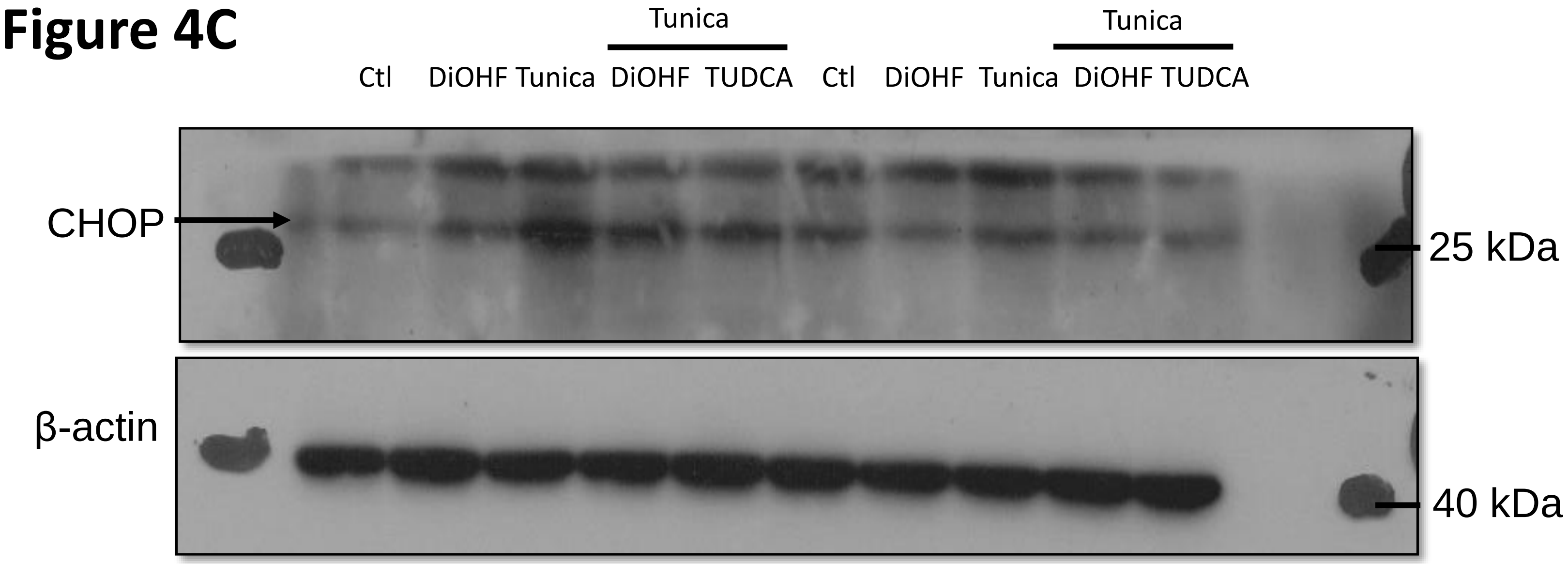


**Figure 4D**

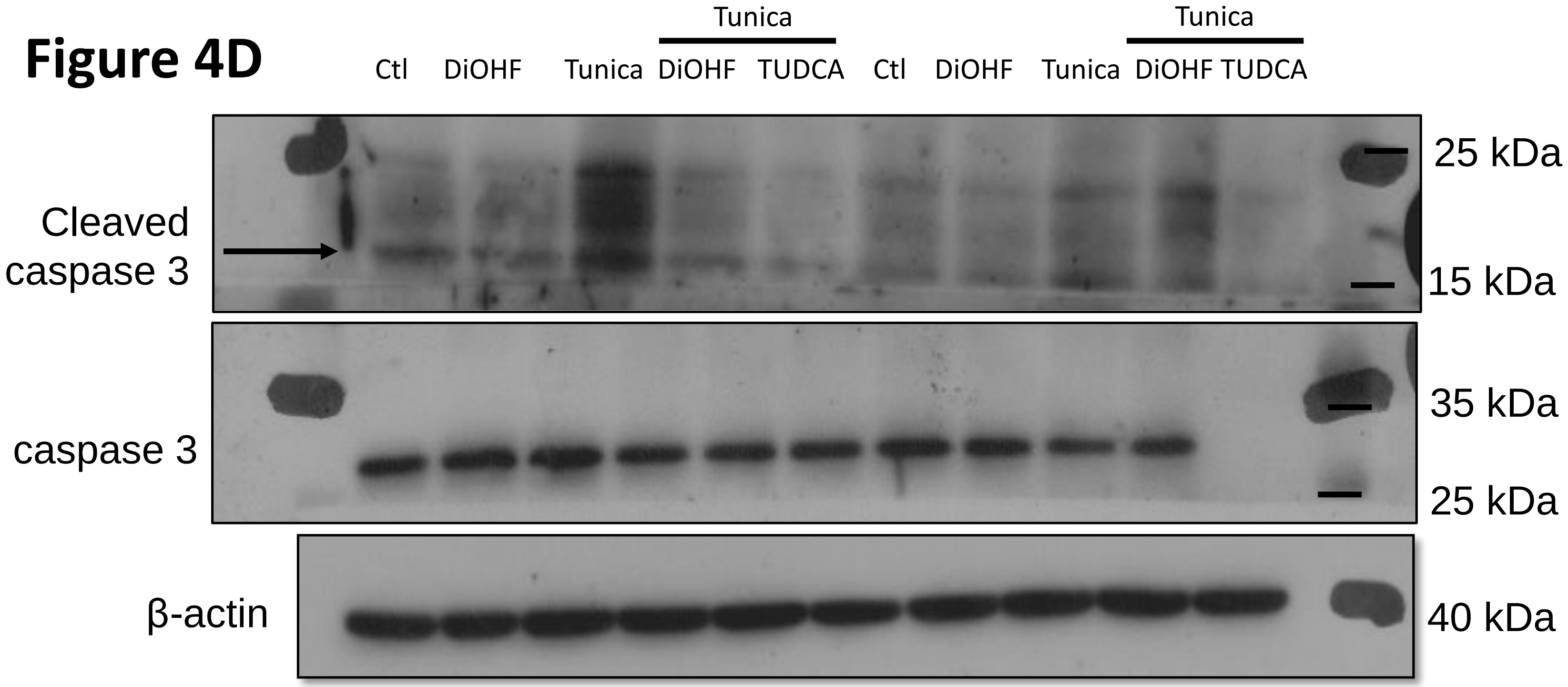

Figure 4E

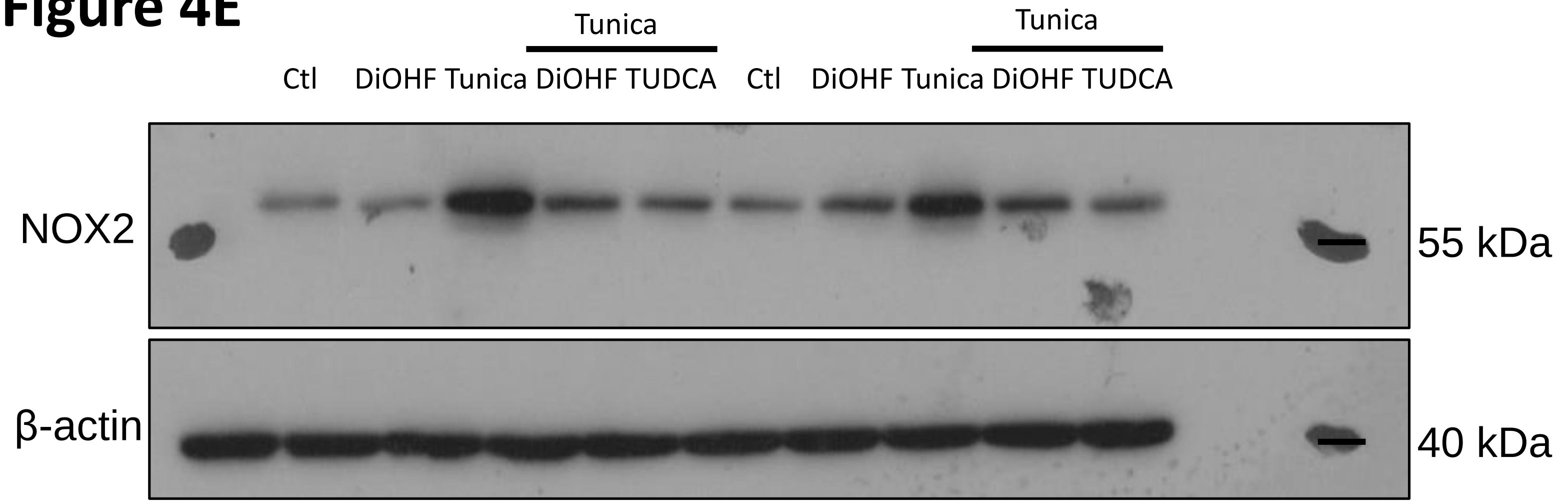


Figure 4F

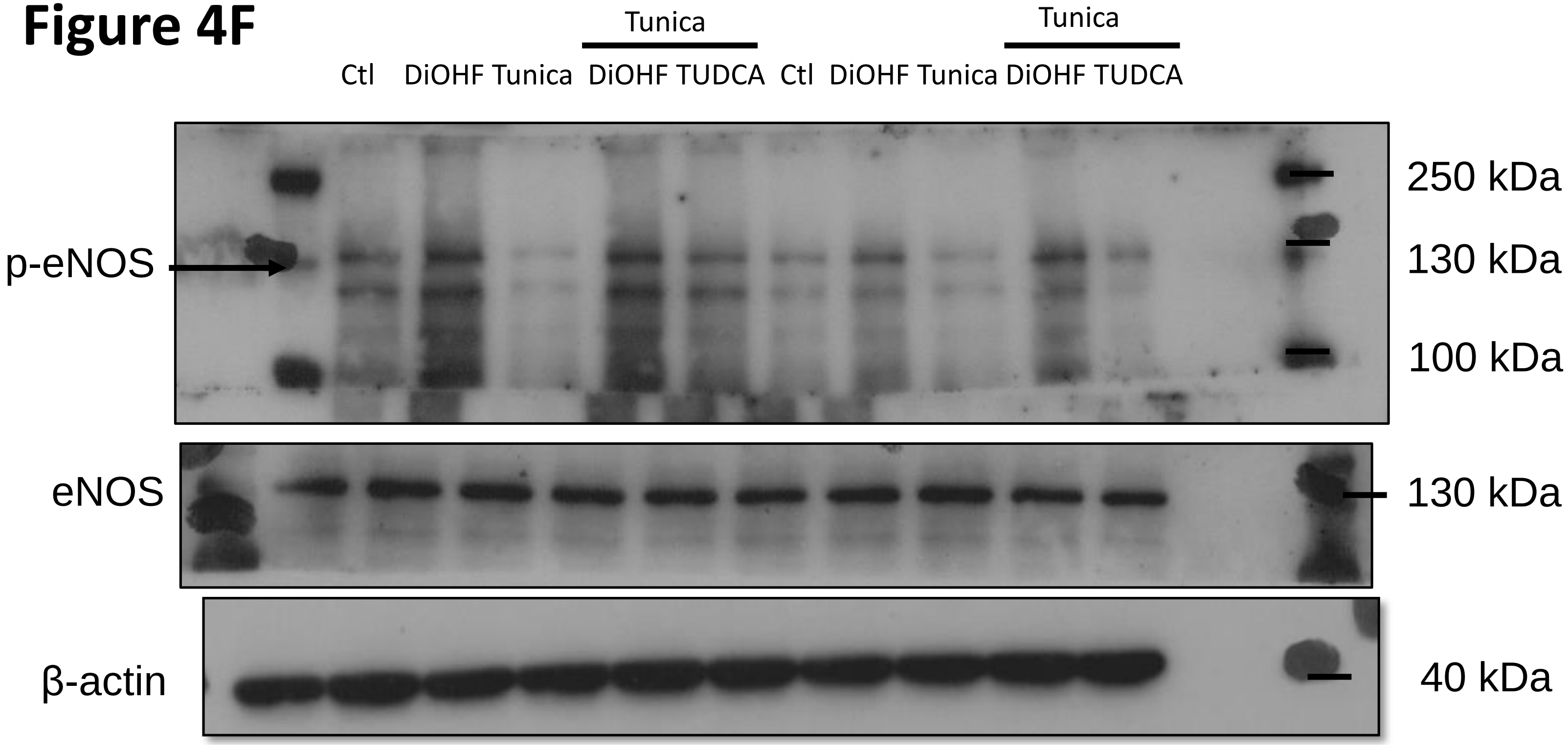

# Figure 5A

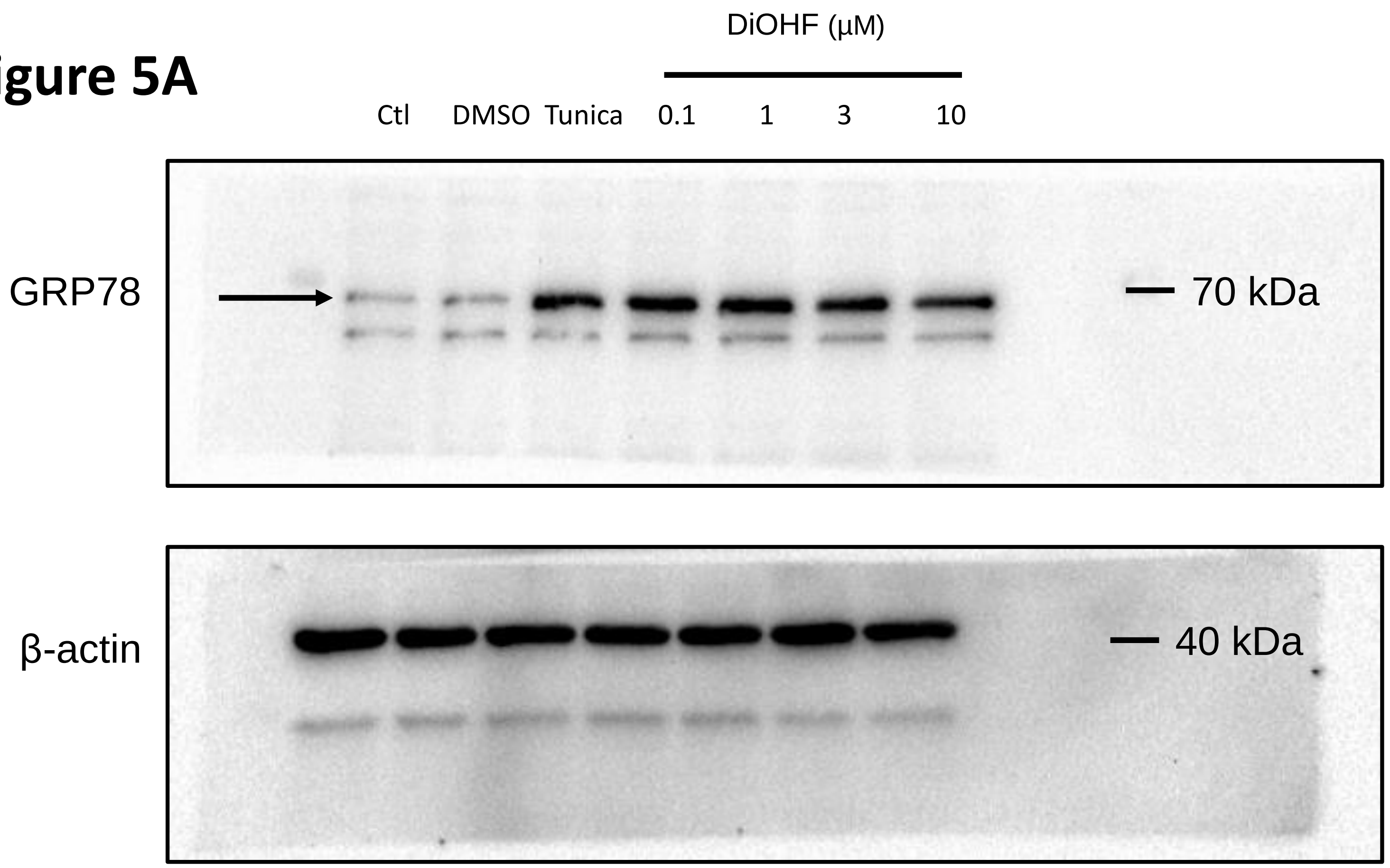

Figure 5B

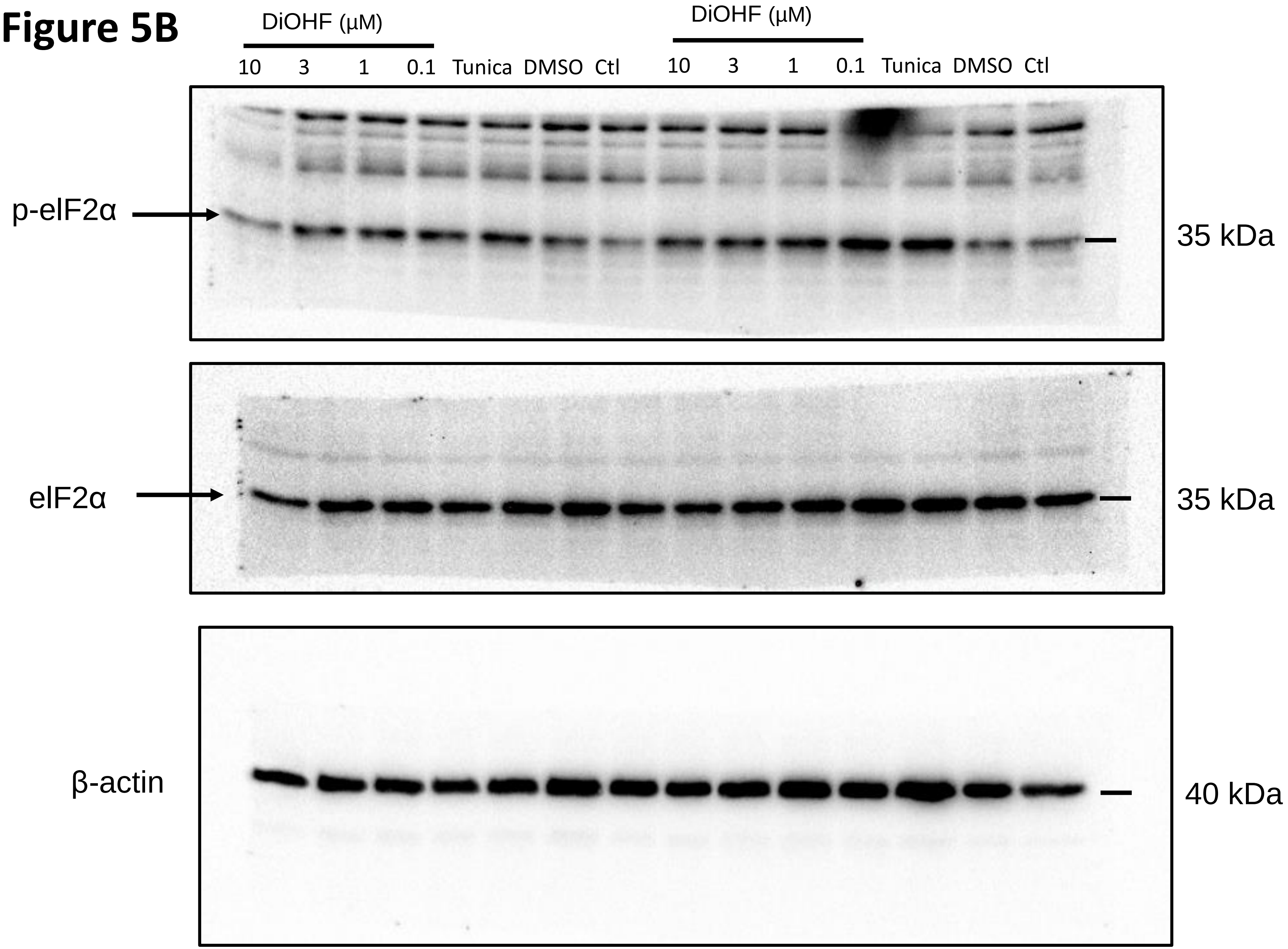
DiOHF (μM)
10
3
1
0.1
Tunica
DMSO
Ctl
DiOHF (μM)
10
3
1
0.1
Tunica
DMSO
Ctl
p-eIF2α
35 kDa
eIF2α
35 kDa
β-actin
40 kDa
